# Characterization of bleeding in thrombotic thrombocytopenic purpura in the precaplacizumab era: a retrospective nationwide analysis

**DOI:** 10.1016/j.rpth.2024.102654

**Published:** 2024-12-12

**Authors:** Amir A. Mahmoud, Mariam Mostafa, Ali Abdelhay, Mouhamed Yazan Abou-Ismail, Shruti Chaturvedi

**Affiliations:** 1 Division of Hematology, Department of Medicine, Washington University School of Medicine in St. Louis, Missouri, USA; 2 Department of Internal Medicine, Rochester General Hospital, Rochester, New York, USA; 3 Division of Hematology and Hematologic Malignancies, Department of Internal Medicine, University of Utah Health Sciences Center, Salt Lake City, Utah, USA; 4 Division of Hematology, Department of Medicine, Johns Hopkins University School of Medicine, Baltimore, Maryland, USA

**Keywords:** bleeding, caplacizumab, National Inpatient Sample, plasma exchange, thrombotic thrombocytopenic purpura

## Abstract

**Background:**

The addition of caplacizumab to immune thrombotic thrombocytopenia (iTTP) treatment options has led to a renewed interest in characterizing the epidemiology and risk factors for bleeding in iTTP. Limited data exist on the bleeding risk in iTTP due to systemic underreporting in earlier cohorts.

**Objectives:**

To describe the incidence, patterns, and predictors of bleeding in hospitalized iTTP patients independent of caplacizumab use.

**Methods:**

We retrospectively analyzed the National Inpatient Sample database (2012-2019) and identified adult patients with a diagnosis of iTTP. Predictors of bleeding were determined by multivariable logistic regression analysis.

**Results:**

We identified 3103 iTTP hospitalizations; bleeding occurred in 594 (19.1%), and 157 (5.1%) were characterized by major bleeding. Mucocutaneous bleeding (7.6%) was the most frequent type of bleeding and included heavy menstrual bleeding (2.6%), gingival (2.3%), epistaxis (1.4%), and skin/procedure-related bleeding (1.3%). This was followed closely by gastrointestinal bleeding (5.6%). Patients with bleeding were more likely to be Hispanic, have a weekend admission, and have a higher prevalence of comorbidities. In the multivariable analysis, Hispanic race (odds ratio [OR], 1.48; 1.14-1.91), Asian/Pacific Islander/Native American race (OR, 2.04; 1.51-2.76), coronary artery disease (OR, 1.70; 1.38-2.11), heart failure (OR, 1.39; 1.13-1.72), autoimmune disease (OR, 2.61; 2.08-3.26), Charlson Comorbidity Index ≥ 3 (OR, 2.08; 1.66-2.61), weekend admission (OR, 1.45; 1.22-1.72), and delay ≥2 days in plasma exchange initiation (OR, 1.63; 1.38-1.92), were significantly associated with major bleeding.

**Conclusions:**

Bleeding is a relatively common issue in acute iTTP that has not been adequately addressed in existing literature. Further studies are needed to elucidate this risk and associated factors, especially given the incorporation of caplacizumab in the treatment of iTTP.

## Essentials

• Bleeding risk in iTTP is underreported and understudied.
• This study describes bleeding patterns and predictors in iTTP hospitalizations.
• Bleeding occurred in 19.1% of patients, with mucocutaneous bleeding most common.
• Higher bleeding risk is linked to factors like race, comorbidities, and delayed plasma exchange.

## 1 Introduction

Immune-mediated thrombotic thrombocytopenic purpura (iTTP) is a rare life-threatening thrombotic microangiopathy caused by an autoantibody mediated deficiency of ADAMTS-13, the von Willebrand factor (VWF) cleaving protease, resulting in the accumulation of ultra-large VWF multimers that leads to the formation of platelet-rich microthrombi causing end-organ ischemia [1]. The clinical presentation of iTTP is characterized by microangiopathic hemolytic anemia, thrombocytopenia, and variable organ dysfunction.

iTTP management is appropriately focused on addressing thrombotic complications, given their nearly-universally life-threatening nature. The addition of caplacizumab to the iTTP treatment armamentarium has led to a renewed interest in characterizing the epidemiology and risk factors for bleeding in iTTP. Caplacizumab is a nanobody that inhibits VWF-platelet interactions and reduces times to platelet count recovery, as well as the composite of early iTTP recurrence, thromboembolism, and death [2]. However, this comes at the cost of increased bleeding due to its mechanism of action that leads to an effect resembling an acquired type 2M von Willebrand disease [3,4]. In the phase 3 HERCULES trial, 65% of patients in the caplacizumab arm and 48% on placebo experienced mucocutaneous bleeding [2]. As expected, bleeding was increased in the caplacizumab-treated patients, but the bleeding rate in the placebo arm was also surprisingly high, suggesting that bleeding may have been underreported in previous observational studies that did not systematically evaluate for bleeding due to the predominantly thrombotic nature of iTTP complications. Understanding baseline bleeding risk in iTTP is crucial for optimizing therapy since avoiding caplacizumab in patients at increased risk of bleeding is desirable.

Few studies have focused on bleeding in iTTP, and elucidating bleeding risk from published iTTP studies is hampered by heterogeneous methodologies and underreporting. Consequently, a comprehensive characterization of bleeding in iTTP has remained elusive. We used the Nationwide Inpatient Sample (NIS) database to describe the incidence, patterns, and predictors of bleeding during acute iTTP episodes.

## 2 Methods

### 2.1 Data source

We performed a retrospective analysis using the NIS database for the years 2012 to 2019. The NIS database is sponsored by the Agency for Healthcare Research and Quality as part of the Healthcare Cost and Utilization Project. It is the largest all-payer inpatient care database in the United States, containing annual data on >7 million hospital stays. NIS identifies diseases with the International Classification of Diseases, 9th Revision, Clinical Modification (ICD-9-CM) code for diagnosis for 2012 up to the first three-quarters of 2015 (from January to September); and ICD-10-CM codes for the last quarter (from October to December) of 2015 and onward.

### 2.2 Study population

We used the NIS database to identify all patients aged ≥18 years with a diagnosis of thrombotic microangiopathy who were discharged between the years 2012 and 2019. We used ICD-9-CM code 446.6 and ICD-10-CM code M31.1 to identify a diagnosis of thrombotic microangiopathy. We used ICD-9-CM procedure code 99.71 as well as ICD-10-CM procedure codes 6A550Z3 and 6A551Z3 to identify therapeutic plasma exchange (TPE). By limiting the study population to hospitalizations for thrombotic microangiopathy for which TPE was performed, we identified a cohort that represents patients with likely iTTP.

### 2.3 Patient and hospitalization characteristics

The patient characteristics extracted were age, sex, race, and median household income. Hospitalization characteristics of interest were weekend admission, hospital location (urban or rural), and teaching status (academic or nonacademic). The presence of comorbidities (diabetes, obesity, excessive alcohol use, congestive heart failure [CHF], coronary artery disease [CAD], chronic kidney disease or end-stage renal disease, autoimmune diseases, liver diseases, and cancer), pregnancy or delivery, long-term outpatient aspirin or anticoagulant use, and blood and platelet transfusions were identified using their respective ICD-9-CM and ICD-10-CM codes for diagnosis or procedure (see Supplementary Methods). Similarly, comorbidities were identified using ICD-9-CM and ICD-10-CM codes and subsequently weighted to calculate the Charlson Comorbidity Index (CCI), following the methodology outlined by Lix et al. [5]. Due to the limitations of the NIS database, a look-back period was not applied. We also extracted the time duration in days between admission and initiation of plasmapheresis.

### 2.4 Bleeding

We identified bleeding events including gastrointestinal (GI) bleeding (upper, lower, and unspecified), hemoptysis, central nervous system (CNS) bleeding (subarachnoid hemorrhage, intracerebral hemorrhage, and subdural hemorrhage), orbital or retinal hemorrhage, hemoperitoneum, hemothorax or hemopericardium, heavy menstrual bleeding or menorrhagia, gingival or buccal bleeding, skin wound or procedure-related bleeding, hematuria, and any percutaneous, endoscopic, or surgical intervention for bleeding using respective ICD-9 and ICD-10 codes. ICD-10 codes for bleeding outcomes have shown good diagnostic accuracy in different validation studies, ranging between 81% and 95% [[6], [7], [8]]. We included codes in primary and secondary positions given the study population being patients primarily admitted for TTP. This was demonstrated by Shehab et al. [8] to improve sensitivity while maintaining adequate predictive value. We also stratified severity into 3 categories (clinically nonrelevant nonmajor bleeding, clinically relevant nonmajor bleeding, and major bleeding) utilizing the International Society on Thrombosis and Haemostasis (ISTH) definitions for bleeding severity [9,10]. All ICD-9 and ICD-10 codes for bleeding events and criteria for severity are described in the Supplementary Material.

### 2.5 Statistical analysis

Baseline patient and hospital characteristics were summarized using descriptive statistics and compared between iTTP patients with and without bleeding. Categorical and continuous variables were first compared using the chi-squared test and Mann–Whitney U-test, respectively. Subsequently, separate multivariable logistic regression models were developed to identify risk factors for bleeding, major bleeding, and mortality. Covariates included in the multivariable models were selected based on clinical plausibility and statistical significance. NIS discharge weights were applied to multivariable analyses as recommended by the Healthcare Cost and Utilization Project to ensure robustness and that the results are representative of the national population. Two-sided *P* < .05 was considered significant. We did not correct for multiplicity in our analysis. All statistical analyses were performed using IBM SPSS statistics version 26.

## 3 Results

### 3.1 Cohort characteristics and bleeding rate

Between 2012 and 2019, the NIS database included 3103 presumed iTTP patients aged 18 or above who underwent TPE during their hospital stay. Their median age was 49 years (range, 18-90 years) and 65.9% were women. Data on race were missing for 146 (4.7%) entries. Bleeding occurred in 594 (19.1%) of all presumed iTTP hospitalizations with a total of 707 bleeding events. Patients with and without bleeding complications were not significantly different in terms of age (48.8 vs 48.6 years; *P* = .759), female sex (67.3% vs 65.6%; *P* = .41), income quartiles (*P* = .62), pregnancy status (1.3% vs 2.2%; *P* = .189), long-term outpatient use of aspirin (5.9% vs 6.2%; *P* = .79) or anticoagulants (4.2% vs 3.5%; *P* = .41), and hospital teaching status (*P* = .79). Patients with bleeding were more likely to be Hispanic (11.8% vs 8.8%; *P* = .038) and to be admitted over a weekend (24.9% vs 20.9%; *P* = .038) than those without bleeding. Baseline patient and hospital characteristics are delineated in Table 1. Most comorbidities as well as high CCI scores were more prevalent in the bleeding groups except for mild-to-moderate liver disease and obesity.

**Table 1:** Characteristics in thrombotic thrombocytopenic purpura hospitalizations with and without bleeding.

Variable	iTTP hospitalizations (*n* = 3103)		
	With bleeding (*n* = 594)	Without bleeding (*n* = 2509)	*P* value
**Age, mean, (y)**	48.8	48.6	.759
**Female, *n* (%)**	400 (67.3)	1645 (65.6)	.412
**Race, *n* (%)**			.038
Caucasian	259 (45.0)	1054 (44.2)	
African American	213 (37.0)	1000 (42.0)	
Hispanic	68 (11.8)	210 (8.8)	
Others	35 (6.1)	118 (5.0)	
**Income quartile, *n* (%)**			.623
1	201 (34.8)	863 (35.1)	
2	140 (24.3)	597 (24.3)	
3	139 (24.1)	541 (22.0)	
4	97 (16.8)	458 (18.6)	
**Teaching status of the hospital, *n* (%)**			.690
Rural hospital	4 (0.7)	13 (0.5)	
Urban nonteaching	78 (13.1)	359 (14.3)	
Urban teaching	512 (86.2)	2137 (85.2)	
**Weekend admission, *n* (%)**	148 (24.9)	525 (20.9)	.034
**Pregnancy/delivery, *n* (%)**	8 (1.3)	55 (2.2)	.189
**Comorbidities, *n* (%)**			
Coronary artery disease	84 (14.1)	278 (11.1)	.037
Congestive heart failure	98 (16.5)	267 (10.6)	<.001
Autoimmune disease	60 (10.1)	162 (6.5)	.002
Chronic/End-stage renal disease	160 (26.9)	557 (22.2)	.014
Mild-to-moderate liver disease	24 (5.7)	174 (6.9)	.289
Severe liver disease	23 (3.9)	40 (1.6)	<.001
Cancer (localized or metastatic)	74 (12.5)	215 (8.6)	.003
Excessive alcohol use	33 (5.6)	92 (3.7)	.035
Obesity	102 (17.2)	445 (17.7)	.745
**Charlson Comorbidity Index, *n* (%)**			<.001
0	148 (24.9)	911 (36.3)	
1	119 (20.0)	505 (20.1)	
2	96 (16.2)	365 (14.5)	
≥3	231 (38.9)	728 (29.0)	
**Long-term aspirin use, *n* (%)**	35 (5.9)	155 (6.2)	.794
**Long-term anticoagulant use, *n* (%)**	25 (4.2)	88 (3.5)	.412
**Time to initiation of plasma exchange (d), mean (95% CI)**	3.8 (3.3-4.4)	2.3 (2.1-2.5)	<.001
**Outcomes**			
In-hospital mortality, *n* (%)	100 (16.8)	171 (6.8)	<.001
Length of stay (d), mean (95% CI)	19.9 (18.3-21.5)	14.0 (13.4-14.5)	<.001
Received blood transfusion, *n* (%)	290 (48.8)	842 (33.6)	<.001
Received platelet transfusion, *n* (%)	126 (21.2)	307 (12.2)	<.001

### 3.2 Characterization of bleeding episodes

**Figure 1:**
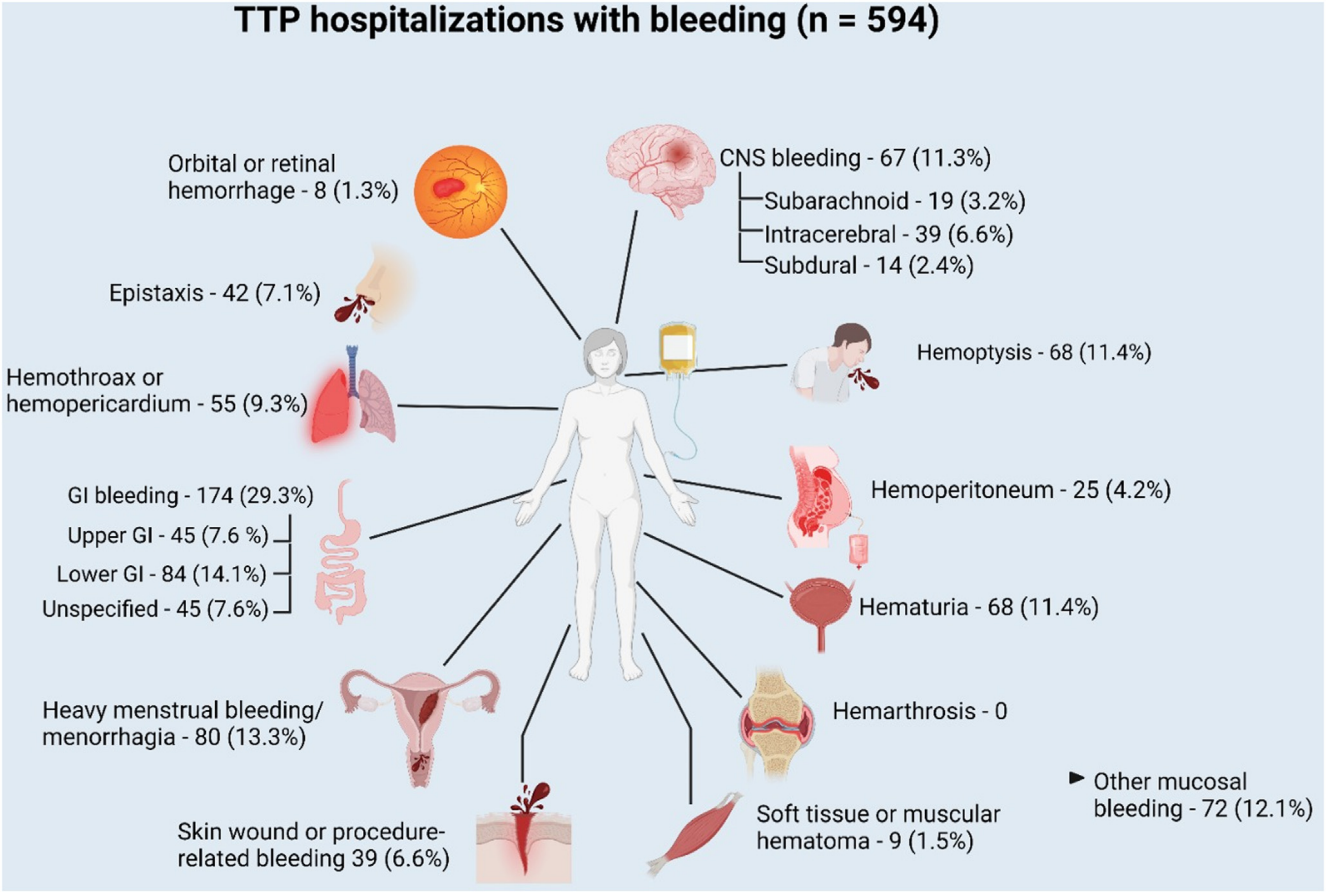
Frequency of different bleeding types and percentage among thrombotic thrombocytopenic purpura hospitalizations with bleeding.

Mucocutaneous bleeding was the most frequent type of bleeding (7.6%; Table 2) and included heavy menstrual bleeding or menorrhagia in 80 (2.6%), gingival or buccal bleeding or ecchymosis in 72 (2.3%), epistaxis in 42 (1.4%), and skin or procedure-related bleeding in 39 (1.3%). This was followed closely by GI bleeding in 174 (5.6%), with lower GI bleeding being more common than upper (2.7% vs 1.5%), then hemoptysis in 68 (2.2%), hematuria in 68 (2.2%), and CNS bleeding in 67 (2.2%). There were no events of hemarthrosis and only 9 (0.3%) events of soft tissue or muscle hematoma. Thirty-seven (1.2%) of the hospitalizations with bleeding required percutaneous, endoscopic, or surgical intervention for bleeding. Figure 1 summarizes frequency and patterns of bleeding in acute iTTP.

**Table 2:** Types of bleeding in TTP hospitalizations with bleeding.

Type of bleeding	*N* (% of all hospitalizations)
TTP hospitalizations with any bleeding	594 (19.1)
GI Bleeding	174 (5.6)
Upper GI bleeding	45 (1.5)
Lower GI bleeding	84 (2.7)
Unspecified	45 (1.5)
Hemoptysis	68 (2.2)
CNS bleeding	67 (2.2)
Subarachnoid hemorrhage	19 (0.6)
Intracerebral hemorrhage	39 (1.3)
Subdural hemorrhage	14 (0.5)
Orbital or retinal hemorrhage	8 (0.3)
Hemoperitoneum	25 (0.8)
Hemothorax or Hemopericardium	55 (1.8)
Hemarthrosis	0
Epistaxis	42 (1.4)
Heavy menstrual bleeding/menorrhagia	80 (2.6)
Other mucosal bleeding (gingival/buccal or ecchymotic)	72 (2.3)
Skin wound or procedure-related bleeding	39 (1.3)
Soft tissue or muscular hematoma	9 (0.3)
Hematuria	68 (2.2)

CNS, central nervous system; GI, gastrointestinal; TTP, thrombotic thrombocytopenic purpura.

Red blood cell transfusions were administered in 1132 (36.5%) (27 [0.9%] with ≥ 2 transfusions during hospital stay) and platelet transfusions in 433 (14.0%) of hospitalizations for presumed acute iTTP. More patients in the bleeding group (Table 1) received blood transfusions (48.8% vs 33.6%; *P* < .001) and platelet transfusions (21.2% vs 12.2%; *P* < .001). Time to initiation of TPE was delayed in the bleeding group (mean, 3.8 vs 2.3 days; *P* < .001) and patients who received platelet transfusion (4.2 vs 2.3 days; *P* < .001).

**Figure 2:**
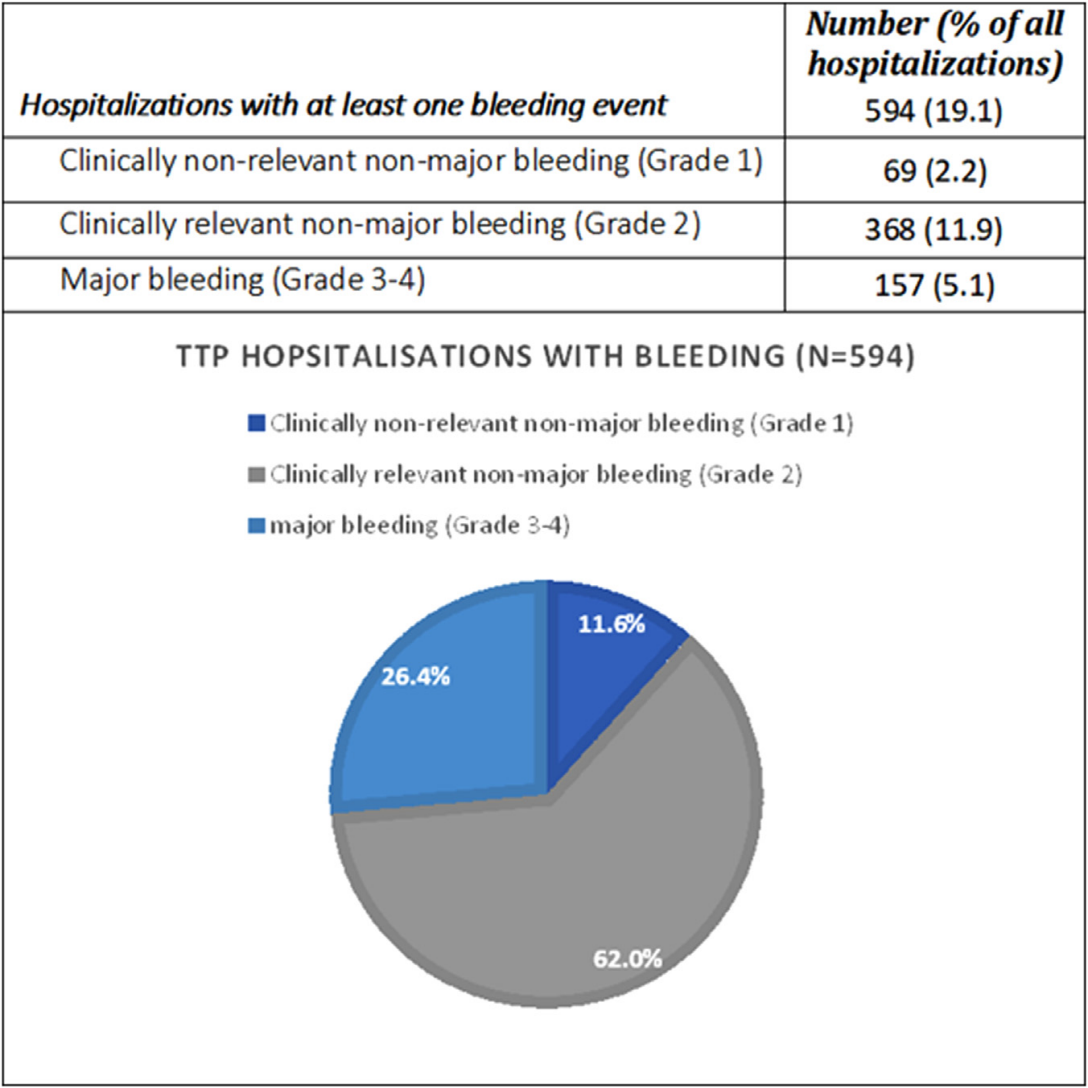
Bleeding severity in thrombotic thrombocytopenic purpura hospitalizations.

We stratified the 594 hospitalizations with bleeding according to bleeding severity (Figure 2). We identified 69 (2.2%) with clinically nonrelevant nonmajor bleeding (grade 1), 368 (11.9%) with clinically relevant nonmajor bleeding (grade 2), and 157 (5.1%) with major bleeding (grade 3-4) events. Among patients with major bleeding (Supplementary Table S1), CNS bleeding was the most common at 67 (42.7%), followed by hemothorax or hemopericardium in 55 (35.0%), and hemoperitoneum in 25 (15.9%). Baseline characteristics of iTTP patients who developed major bleeding compared with those who did not are delineated in Supplementary Table S2.

#### 3.2.1 Sensitivity analysis

As caplacizumab was approved in 2019, there might be a risk that bleeding was influenced by caplacizumab use in our study population, even though widespread use is likely to be limited early on. We repeated the analysis for bleeding events omitting hospitalization for patients discharged in 2019 from our study population. Out of 2769 hospitalizations, we found a similar number of bleeding events 510 (18.4%). Additionally, there was a similar number of major bleeding events 132 (4.8%), clinically nonrelevant nonmajor bleeding 61 (2.2%), and clinically relevant nonmajor bleeding 317 (11.4%).

### 3.3 Predictors of bleeding

**Figure 3:**
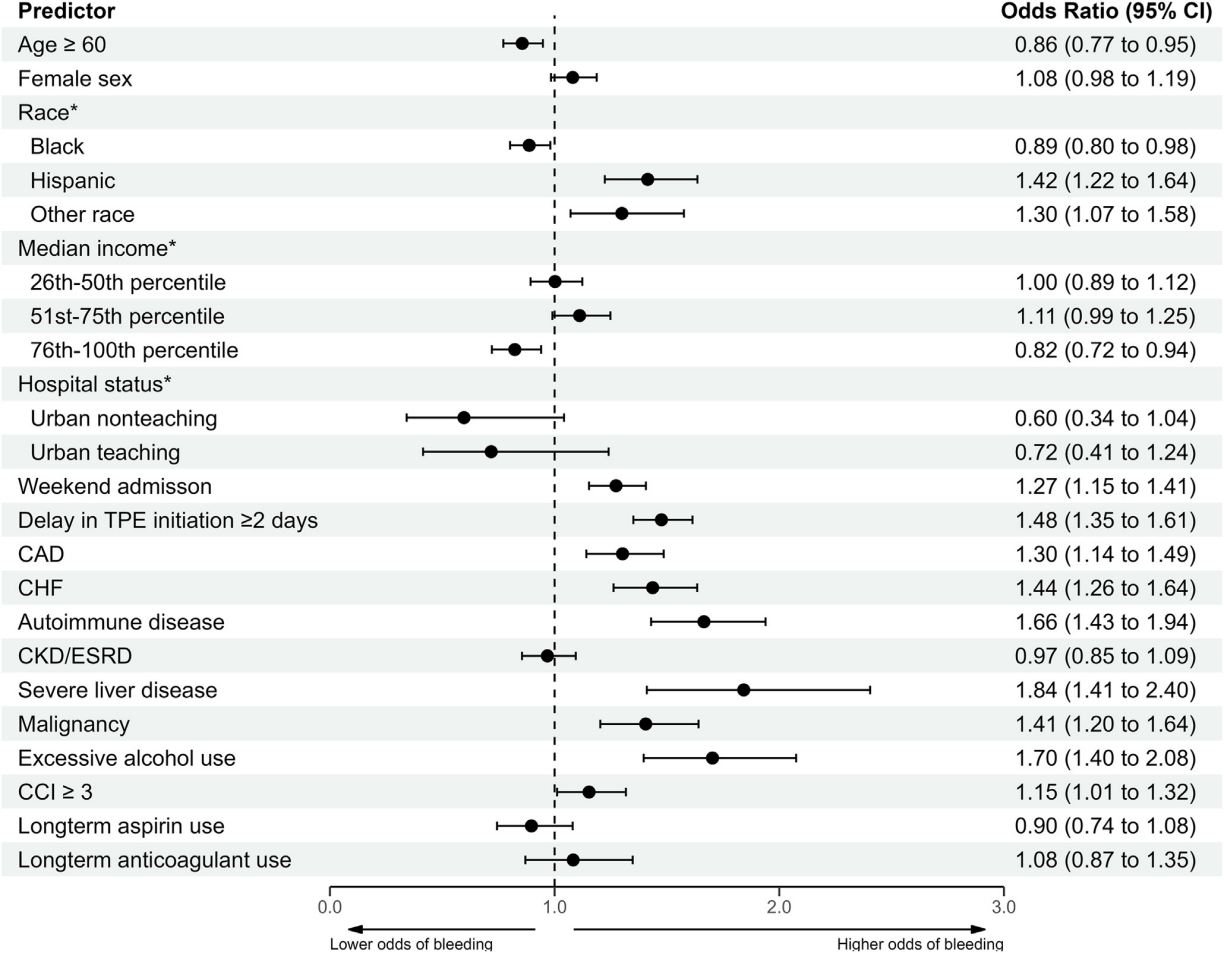
Predictors of bleeding in thrombotic thrombocytopenic purpura hospitalizations. Multivariable binary logistic regression analysis for the outcome of any bleeding in the hospitalization. ∗Reference variables were white race, 0 to 25th percentile, and rural for race, median income, and hospital status, respectively.

A multivariable logistic regression analysis was performed to identify risk factors for any bleeding in TTP patients (Figure 3), adjusting for age, sex, race, median household income, hospital status, weekend admission, delay in TPE initiation, comorbidities, CCI ≥ 3, and long-term outpatient aspirin and anticoagulant use. Variables significantly associated with bleeding in TTP hospitalizations were Hispanic race (adjusted odds ratio (OR), 1.42; 95% CI, 1.22-1.64), weekend admission (OR, 1.27; 95% CI, 1.15-1.41), delay in TPE initiation (OR, 1.48; 95% CI, 1.35-1.61), CHF (OR, 1.44; 95% CI, 1.26-1.64), autoimmune disease (OR, 1.66; 95% CI, 1.43-1.94), severe liver disease (OR, 1.84; 95% CI, 1.41-2.40), malignancy (OR, 1.41; 95% CI, 1.20-1.64), excessive alcohol use (OR, 1.70; 95% CI, 1.40-2.08), and CCI ≥ 3 (OR, 1.15; 95% CI, 1.01-1.32).

**Figure 4:**
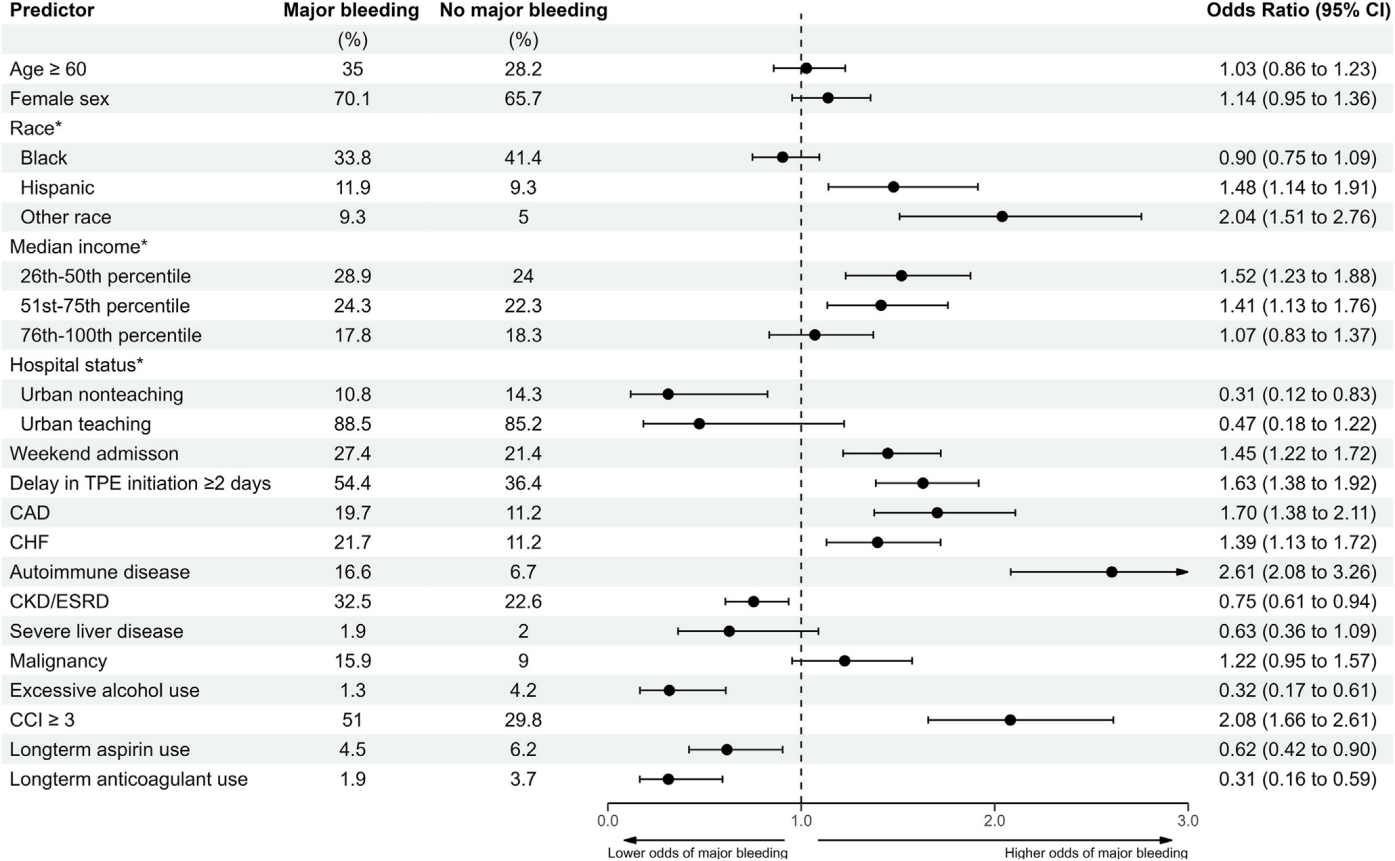
Predictors of major bleeding in thrombotic thrombocytopenic purpura hospitalizations. Multivariable binary logistic regression analysis for the outcome of major bleeding in the hospitalization. ∗Reference variables were white race, 0 to 25th percentile, and rural for race, median income, and hospital status, respectively.

A similar multivariable analysis was performed to identify risk factors for major bleeding (Figure 4). Variables significantly associated with higher odds of major bleeding in TTP hospitalizations were Hispanic race, Asian/Pacific Islander or Native American race (compared to white), income 25th to 75th percentile, CAD, CHF, underlying autoimmune disease, CCI score ≥ 3, weekend admission and delay ≥ 2 days in TPE initiation. Given the concern that the respective ICD codes for long-term aspirin and anticoagulant use may not capture medication history adequately, we repeated the analysis for major bleeding without adjusting for the aforementioned 2 variables, with similar results and estimates (Supplementary Figure S2). A separate multivariable analysis was performed to identify predictors of mortality in the current population (Supplementary Figure S1). Age ≥ 60 years, weekend admission, delay ≥ 2 in initiation of TPE, CHF, underlying autoimmune disease, severe liver disease, malignancy, major bleeding, blood transfusion, and platelet transfusion were independently associated with mortality.

## 4 Discussion

iTTP is unquestionably a severe thrombotic disorder. To our knowledge, our study is the first to characterize bleeding risk in iTTP, independent of VWF-targeted therapies, in a large, real-world cohort. This is particularly relevant in the current iTTP treatment landscape, where understanding the baseline bleeding risk in iTTP could help tailor caplacizumab use by identifying patients at increased risk of bleeding.

In this large cohort primarily from the precaplacizumab era, almost a fifth of presumed iTTP hospitalizations (19.1%) had at least one bleeding event and 5.1% were characterized by major bleeding. Earlier iTTP cohorts have reported a wide range of bleeding rates from 0% to 54% [[11], [12], [13], [14]]. The landmark study by Rock et al. [14] in 1991 comparing TPE and plasma infusion reported a bleeding rate of 7.8% in the study population, which received concomitant aspirin and dipyridamole. A retrospective analysis of the Spanish iTTP registry from 2002 to 2021 found bleeding rates of 59% in patients with an initial episode of iTTP [15]. More recently, an integrated analysis of the HERCULES and TITAN trials on the use of caplacizumab for the treatment of iTTP also reported a high bleeding rate (47%) in iTTP patients not treated with caplacizumab [16]. Comparisons in real-world data of caplacizumab use are often limited to historic cohorts with systematic underreporting of bleeding events, hindering the ability to accurately characterize the difference in bleeding risk [17,18].

Bleeding in iTTP is likely driven by disordered primary hemostasis due to profound thrombocytopenia. Like other disorders of primary hemostasis, mucocutaneous bleeding was the most common type of bleeding in iTTP. Interestingly, there was a high rate of GI bleeding (5.6% of hospitalizations) representing 24.6% of all bleeding events. This is higher than reported in the study by Rock et al. [14] (6.9%) and in an analysis of 72 iTTP episodes from the Australian iTTP registry (4%) that reported cutaneous bleeding in 39%, menorrhagia in 10%, CNS bleeding in 3%, and pulmonary hemorrhage in 1% of episodes [11]. In contrast, no GI bleeding events were noted in the control group of the HERCULES trial, where catheter or puncture site-associated bleeding was the most common bleeding event [2].

The limited data on bleeding from previous studies suggest that most bleeding events in iTTP are mild to moderate in severity but cases of fatal GI and CNS bleeding have been reported in observational studies and clinical trials of iTTP [11,14,16,19,20]. For example, serious bleeding-related adverse events from cerebral hemorrhage and hematuria were reported in 2 (5%) patients in the control group of the TITAN trial, while one (1%) patient in the HERCULES trial developed a hemorrhagic transformation of stroke. In our cohort, there were 157 episodes of major bleeding (5.1% of iTTP episodes), and major bleeding was independently associated with mortality. Of all major bleeding events, CNS bleeding was the most common (42.7%) followed by hemothorax or hemopericardium (35%). We also identified 13 events each of GI bleeding and hemoptysis, that were classified as severe due to the need for blood transfusions (≥2).

In comparison with data on bleeding with caplacizumab use, our study reported similar rates of grade 2-4 bleeding (17%) compared to the triplet regimen study by Coppo et al. [18] utilizing caplacizumab alongside standard of care (18.9%). In a large international retrospective analysis (the Capla 500 project) that reviewed 942 iTTP patients who received a regimen containing caplacizumab, major bleeding was identified in 19 (2.2%), clinically relevant nonmajor bleeding in 34 (3.7%), and nonclinically relevant nonmajor bleeding in 114 (14%) [21]. The bleeding rates in this analysis are surprisingly low. It is possible that lower bleeding rates were seen due to patient selection at highly experienced iTTP centers based on clinically estimated bleeding risk. However, since this was a retrospective study, it is also likely that bleeding, at least nonmajor bleeding, may have been underreported due to incomplete documentation. Given our study’s reliance on ICD coding, the overall incidence of nonmajor bleeding is likely underestimated due to underreporting. However, underreporting is less expected with major bleeding events.

There are no prior studies assessing predictors of bleeding risk in iTTP. In our study, CCI ≥ 3, delay ≥ 2 days in TPE initiation, weekend admission, CAD, CHF, underlying autoimmune disease, income 25th to 75th percentile, and race (Hispanic, Asian or Pacific Islander or Native American), were significantly associated with major bleeding. Long-term outpatient aspirin or anticoagulant use was not associated with increased bleeding risk, although it is likely that these medications may not have been continued during the hospitalization due to thrombocytopenia and the need for procedures. Further, due to the lack of medication data in the NIS, we relied on ICD-9 or ICD-10 codes of long-term anticoagulant and aspirin use which may not capture medication history accurately. Delays in TPE initiation were associated with platelet transfusions; it is possible that this reflects patients presenting with bleeding in whom diagnosis was also delayed rather than a causative relationship of treatment delays with bleeding. Older age (≥60 years) was not associated with major bleeding in our study though a previous analysis of the NIS database, which focused on the impact of age on the delays in initiation of TPE for iTTP, reported marginal significance of age ≥ 60 years as a risk factor on major bleeding [22]. This discrepancy is likely due to the different population size (2992 vs 3103, respectively), since the prior analysis excluded entries with missing data on timing of TPE. A different classification of bleeding severity was also used in the previous study, likely altering the number of bleeding events per age group. Our results suggest that multimorbidity increases major bleeding risk. Additional, ideally prospective, studies are needed to establish predictors of bleeding in iTTP patients, which may guide clinicians to tailor therapy according to bleeding risk.

A surprising finding of our study was that 36.5% and 14% of presumed iTTP hospitalizations included red cell and platelet transfusions, respectively. Platelet transfusions are generally avoided in iTTP due to concerns regarding increased rate of thrombotic events and mortality in patients receiving platelet transfusions [23,24]. However, platelet transfusions may be necessary in patients with major or life-threatening bleeding or an urgent need for invasive procedures. Platelet transfusion was independently associated with mortality, even after adjusting for major bleeding. We cannot establish a causative relationship since we cannot ascertain the relative timing of platelet transfusion in relation to making an iTTP diagnosis or a major bleeding event. However, since initiation of TPE was delayed in the platelet transfusion group (mean, 4.2 vs 2.3 days), it is possible that delays in iTTP diagnosis may be contributing to the high number of platelet transfusions in a subset of patients who were initially thought to have thrombocytopenia from a cause other than iTTP.

This study has several limitations, predominantly related to the use of data from a population-based database using diagnostic codes. There is a risk of misclassification bias as we cannot confirm iTTP diagnosis by ADAMTS-13 activity levels, which are not available in this dataset. Previous studies and audits have demonstrated the sensitivity and specificity in using ICD codes to study outcomes [25]. To increase specificity, we only analyzed iTTP episodes treated with TPE. While direct validation of bleeding outcomes within the NIS is limited, prior studies using administrative datasets, including the NIS, have successfully identified bleeding complications across a range of conditions [26,27]. Prior studies have demonstrated the utility of ICD codes in capturing bleeding events, particularly when carefully selected and applied in large cohort studies [8,28]. Nonetheless, misclassification and underreporting remain a potential limitation. We could not identify fatal bleeding in the definition of major bleeding, as data on the direct cause of death are unavailable in the NIS database. The retrospective nature of the study is another limitation. However, this study included 8 years of nationally representative data from the largest available all-payer hospitalization database allowing us to pool a huge number of iTTP hospitalizations to study outcomes in a rare disease, which would have been difficult to do in a prospective manner.

In conclusion, our study demonstrates that bleeding in iTTP patients is a relatively common complication that has not been adequately addressed in existing literature. Further studies are needed to elucidate its incidence and associated risk factors, which may inform clinical decisions, particularly for selecting patients who should or should not receive caplacizumab.

## Funding

S.C. is supported by National Institutes of Health’s National Heart Lung and 10.13039/100013114Blood Institute grants (K99HL150594 and R00HL172303) and an ASH Scholar Award.
