# Supplementary material for: Characterization of bleeding in thrombotic thrombocytopenic purpura in the precaplacizumab era: a retrospective nationwide analysis
Source: Res Pract Thromb Haemost. 2024 Dec 12;9(1):102654. doi: 10.1016/j.rpth.2024.102654 (PMC11742298; doi:10.1016/j.rpth.2024.102654)
Supplement: Supplementary material [file mmc1.docx]

**Categories of bleeding (definitions)**

Clinically non-relevant non-major bleeding (Grade 1)

| **Either:**   1. Other mucosal bleeding (gingival, petechiae, ecchymosis)   or   1. Skin wound/procedure-related bleeding   **AND:**  No other type of bleeding  **AND**:  Transfusion requirement < 2 PRBC per hospitalization |
| --- |

Clinically relevant non-major bleeding (Grade 2)

| **Any:**   1. Epistaxis   or   1. GI bleeding   or   1. Hematuria   or   1. Vaginal bleeding   or   1. Pulmonary bleeding   or   1. Soft tissue/muscle hematoma   or   1. Hemarthrosis   **AND:**  Transfusion requirement < 2 PRBCs per hospitalization  **AND:**  No other type of bleeding |
| --- |

Major bleeding (Grade 3-4)

| ICD9 | **Either:**   1. Any bleeding type from Garde 1-2 bleeding (see above)   **AND**  Transfusion requirement ≥ 2 PRBCs per hospitalization  **Or:**   1. Bleeding in critical location:   -Hemoperitoneum  or  -Hemothorax or Hemopericardium  or  -CNS bleeding  or  -Retinal or orbital hemorrhage |
| --- | --- |

**ICD9/10 codes for bleeding**

Hematuria

| ICD9 | 59971 (gross hematuria) |
| --- | --- |
| ICD10 | R310 (Gross hematuria) |

Epistaxis

| ICD9 | 7847 (epistaxis) |
| --- | --- |
| ICD10 | R040 (epistaxis) |

HMB/menorrhagia

| ICD9 | 6269 (MENSTRUAL DISORDER NOS)  6262 (EXCESSIVE MENSTRUATION) |
| --- | --- |
| ICD10 | N920 (Excessive and frequent menstruation with regular cycle)  N921 (Excessive and frequent menstruation with irregular cycle)  N938 (Other specified abnormal uterine and vaginal bleeding)  N939 (Abnormal uterine and vaginal bleeding, unspecified) |

Other Mucosal Bleeding (gingival/buccal, petechiae, ecchymosis)

| ICD9 | 7848 (Hemorrhage from throat) or 5238 (periodontal disease NEC) or 7827 (Spontaneous ecchymosis) |
| --- | --- |
| ICD10 | R041 (Hemorrhage from throat) or K068 (Other specified disorders of gingiva and edentulous alveolar ridge) or R233 (Spontaneous ecchymosis) or R0489 (Hemorrhage from other sites in respiratory passages) |

Skin wound/procedure-related

| ICD9 | 99811 (Hemorrhage as a procedural complication) or 99812 (HEMATOMA PROC CX) |
| --- | --- |
| ICD10 | L7602/L7622/L7632 (Intra/post-operative hemorrhage/hematoma/accidental puncture/laceration of skin and subcutaneous tissue complicating a procedure) or T8130XA (wound disruption) |

Pulmonary bleeding

| ICD9 | 78630 (Hemoptysis NOS) or 78639 (Hemoptysis NEC) |
| --- | --- |
| ICD10 | R042 (Hemoptysis) or R0489 (Hemorrhage from other sites in respiratory passages) or R049 (Hemorrhage from respiratory passages, unspecified) |

Soft tissue or muscle hematoma

| ICD9 | 72992 (NONTRAUMA HEMA SOFT TISS) |
| --- | --- |
| ICD10 | M7981 (non-traumatic hematoma of soft tissue) |

Hemoperitoneum

| ICD9 | 56881 (Hemoperitoneum) |
| --- | --- |
| ICD10 | K661 (Hemoperitoneum) |

Hemothorax or hemopericardium

| ICD9 | 8602 (Hemothorax) or 51189 (Hemorrhagic effusion) |
| --- | --- |
| ICD10 | J942 (Hemothorax) or I312 (Hemopericardium, NES) or I313 (Pericardial effusion (noninflammatory)) |

Retinal or orbital hemorrhage

| ICD9 | 37923 (Vitreous hemorrhage) or 36281 (Retinal hemorrhage) or 37632 (ORBITAL HEMORRHAGE) |
| --- | --- |
| ICD10 | H4310/H4311/H4312/H4313 (Vitreous hemorrhage) or H3560/H3561/H3562/H3563 (Retinal hemorrhage) or H05231 / H05232 / H05233 / H05239 (orbital hemorrhage) |

Hemarthrosis

| ICD9 | 71910 (HEMARTHROSIS-UNSPEC) or 71911 (HEMARTHROSIS-SHLDER) or 71912 (HEMARTHROSIS-UP/ARM) or 71915 (HEMARTHROSIS-PELVIS) or 71916 (HEMARTHROSIS-L/LEG) or 71917 (HEMARTHROSIS-ANKLE) or 71918 (HEMARTHROSIS-JT NEC) or 71919 (HEMARTHROSIS-MULT JTS) |
| --- | --- |
| ICD10 | M2500 (Hemarthrosis, unspecified joint) or M25011 (Hemarthrosis, right shoulder) or M25012 (Hemarthrosis, left shoulder) or M25019 (Hemarthrosis, unspecified shoulder) or M25021 (Hemarthrosis, right elbow) or M25022 (Hemarthrosis, left elbow) or M25029 (Hemarthrosis, unspecified elbow) or M25051 (Hemarthrosis, right hip) or M25052 (Hemarthrosis, left hip) or M25059 (Hemarthrosis, unspecified hip) or M25061 (Hemarthrosis, right knee) or M25062 (Hemarthrosis, left knee) or M2506 (Hemarthrosis, unspecified knee) |

GI bleeding

| ICD9 | UGI Bleeding: 5780 (Hematemesis) or 4560 (ESOPHAG VARICES W BLEED) or 45620 (BLEED ESOPH VAR OTH DIS) or 53082 (ESOPHAGEAL HEMORRHAGE) or 53100 (AC STOMACH ULCER W HEM) or 53101 (AC STOMAC ULC W HEM-OBST) or 53120 (AC STOMAC ULC W HEM/PERF) or 53121 (AC STOM ULC HEM/PERF-OBS) or 53200 (AC DUODENAL ULCER W HEM) or 53201 (AC DUODEN ULC W HEM-OBST) or 53220 (AC DUODEN ULC W HEM/PERF) or 53221 (AC DUOD ULC HEM/PERF-OBS) or 53300 (AC PEPTIC ULCER W HEMORR) or 53301 (AC PEPTIC ULC W HEM-OBST) or 53320 (AC PEPTIC ULC W HEM/PERF) or 53321 (AC PEPT ULC HEM/PERF-OBS) or 53400 (AC MARGINAL ULCER W HEM) or 53401 (AC MARGIN ULC W HEM-OBST) or 53420 (AC MARGIN ULC W HEM/PERF) or 53421 (AC MARG ULC HEM/PERF-OBS) or  Lower GI bleeding: 5781 (Melena) or 5693 (RECTAL & ANAL HEMORRHAGE)  5789 (GASTROINTEST HEMORR NOS) |
| --- | --- |
| ICD10 | UGI Bleeding: K920 (Hematemesis) or K2081 (Other esophagitis with bleeding) or K2091 (Esophagitis, unspecified with bleeding) or K2101 (Gastro-esophageal reflux disease with esophagitis, with bleeding) or K2211 (Ulcer of esophagus with bleeding) or K226 (Gastro-esophageal laceration-hemorrhage syndrome) or K250 (Acute gastric ulcer with hemorrhage) or K252 (Acute gastric ulcer with both hemorrhage and perforation) or K260 (Acute duodenal ulcer with hemorrhage) or K262 (Acute duodenal ulcer with both hemorrhage and perforation) or K270 (Acute peptic ulcer, site unspecified, with hemorrhage) or K272 (Acute peptic ulcer, site unspecified, with both hemorrhage and perforation) or K280 (Acute gastrojejunal ulcer with hemorrhage) or K282 (Acute gastrojejunal ulcer with both hemorrhage and perforation) or K2901 (Acute gastritis with bleeding) or K2961 (Other gastritis with bleeding) or K2971 (Gastritis, unspecified, with bleeding) or K2981 (Duodenitis with bleeding) or K2991 (Gastroduodenitis, unspecified, with bleeding) or K31811 (Angiodysplasia of stomach and duodenum with bleeding) or K3182 (Dieulafoy lesion (hemorrhagic) of stomach and duodenum) or I8501 (Esophageal varices with bleeding) or I8511 (Secondary esophageal varices with bleeding) or K762 (Central hemorrhagic necrosis of liver) or  Lower GI bleeding: K921 (Melena) or K51411 (Inflammatory polyps of colon with rectal bleeding) or K51511 (Left sided colitis with rectal bleeding) or K51811 (Other ulcerative colitis with rectal bleeding) or K51911 (Ulcerative colitis, unspecified with rectal bleeding) or K5521 (Angiodysplasia of colon with hemorrhage) or K5701 (Diverticulitis of small intestine with perforation and abscess with bleeding) or K5711 (Diverticulosis of small intestine without perforation or abscess with bleeding) or K5713 (Diverticulitis of small intestine without perforation or abscess with bleeding) or K5721 (Diverticulitis of large intestine with perforation and abscess with bleeding) or K5731 (Diverticulosis of large intestine without perforation or abscess with bleeding) or K5733 (Diverticulitis of large intestine without perforation or abscess with bleeding) or K5741 (Diverticulitis of both small and large intestine with perforation and abscess with bleeding) or K5751 (Diverticulosis of both small and large intestine without perforation or abscess with bleeding) or K5753 (Diverticulitis of both small and large intestine without perforation or abscess with bleeding) or K5781 (Diverticulitis of intestine, part unspecified, with perforation and abscess with bleeding) or K5791 (Diverticulosis of intestine, part unspecified, without perforation or abscess with bleeding) or K5793 (Diverticulitis of intestine, part unspecified, without perforation or abscess with bleeding) or K625 (Hemorrhage of anus and rectum) or K9401 (Colostomy hemorrhage) or K9411 (Enterostomy hemorrhage) or  K922 (Gastrointestinal hemorrhage, unspecified) |

CNS bleeding

| ICD9 | SAH: 430  ICH: 431 or 4329  SDH: 4321  EDH: 4320 |
| --- | --- |
| ICD10 | SAH: I6000 or I6001 or I6002 or I6010 or I6011 or I6012 or I602 or I6020 or I6021 or I6022 or I6030 or I6031 or I6032 or I604 or I6050 or I6051 or I6052 or I606 or I607 or I608 or I609  ICH: I610 or I611 or I612 or I613 or I614 or I615 or I616 or I618 or I619 or 1629  SDH: I6200 or I6201 or I6202 or I6203  EDH: I621 |

Long term aspirin use

| ICD9 | V5866 |
| --- | --- |
| ICD10 | Z7982 |

Long term anticoagulant use

| ICD9 | V5861 |
| --- | --- |
| ICD10 | Z7901 |

Blood transfusion

| ICD9 | 9902/9903/9904 |
| --- | --- |
| ICD10 | 30230H1/30230N1/30233H1/30233N1/30233P1/30240H1/30240N1/30243H1, 30243N1/30250H1/30250N1/30253H1/30260H1/30260N1/30263P1 |

Charlson Comorbidity Index (CCI)

| Comorbid Condition | ICD-9-CM Diagnosis Codes | ICD-10-CM Diagnosis Codes | Weight |
| --- | --- | --- | --- |
| Myocardial Infarction | 410, 412 | I21, I22, I25.2 | 1 |
| Congestive Heart Failure | 398.91, 402.01, 402.11, 402.91, 404.01, 404.03, 404.11, 404.13, 404.91, 404.93, 425.4–425.9, 428 | I09.9, I11.0, I13.0, I13.2, I25.5, I42.0, I42.5–I42.9, I43, I50, P29.0 1 | 1 |
| Peripheral Vascular Disease | 093.0, 437.3, 440, 441, 443.1–443.9, 447.1, 557.1, 557.9, V43.3 | I70, I71, I73.1, I73.8, I73.9, I77.1, I79.0, I79.2, K55.1, K55.8, K55.9, Z95.8, Z95.9 1 | 1 |
| Cerebrovascular Disease | 362.34, 430–438 | G45, G46, H34.0, I60–I69 | 1 |
| Dementia | 290, 294.1, 331.2 | F00–F03, F05.1, G30, G31.1 | 1 |
| Chronic Pulmonary Disease | 416.8, 416.9, 490–505, 506.4, 508.1, 508.8 | I27.8, I27.9, J40–J47, J60–J67 J68.4, J70.1, J70.3 | 1 |
| Connective Tissue Disease/Rheumatic Disease | 446.5, 710.0–710.4, 714.0–714.2, 714.8, 725 | M05, M06, M31.5, M32–M34, M35.1, M35.3, M36.0 | 1 |
| Peptic Ulcer Disease | 531–534 | K25–K28 | 1 |
| Mild Liver Disease | 070.22, 070.23, 070.32, 070.33, 070.44, 070.54, 070.6, 070.9, 570, 571, 573.3, 573.4, 573.8, 573.9, V42.7 | B18, K70.0–K70.3, K70.9, K71.3–K71.5, K71.7, K73, K74, K76.0, K76.2–K76.4, K76.8, K76.9, Z94.4 | 1 |
| Diabetes without Chronic Complications | 250.0–250.3, 250.8, 250.9 | E10.0, E10.1, E10.6, E10.8, E10.9, E11.0, E11.1, E11.6, E11.8, E11.9, E12.0, E12.1, E12.6, E12.8, E12.9, E13.0, E13.1, E13.6, E13.8, E13.9, E14.0, E14.1, E14.6, E14.8, E14.9 | 1 |
| Diabetes with Chronic Complications | 250.4–250.7 | E10.2–E10.5, E10.7, E11.2–E11.5, E11.7, E12.2–E12.5, E12.7, E13.2–E13.5, E13.7, E14.2–E14.5, E14.7 | 2 |
| Paraplegia and Hemiplegia | 334.1, 342, 343, 344.0–344.6, 344.9 | G04.1, G11.4, G80.1, G80.2, G81, G82, G83.0–G83.4, G83.9 | 2 |
| Renal Disease | 403.01, 403.11, 403.91, 404.02, 404.03, 404.12, 404.13, 404.92, 404.93, 582, 583.0–583.7, 585, 586, 588.0. V42.0, V45.1, V56 | I12.0, I13.1, N03.2–N03.7, N052–N05.7, N18, N19, N25.0, Z49.0–Z49.2, Z94.0, Z99.2 | 2 |
| Cancer | 140–172, 174–195.8, 200–208, 238.6 | C00–C26, C30–C34, C37–C41, C43, C45–C58, C60–C76, C81–C85, C88, C90–C97 | 2 |
| Moderate or Severe Liver Disease | 456.0–456.2, 572.2–572.4, 572.8 | I85.0, I85.9, I86.4, I98.2, K70.4, K71.1, K72.1, K72.9, K76.5–K76.7 | 3 |
| Metastatic Carcinoma | 196–199 | C77–C80 | 6 |
| HIV/AIDS | 042–04 | B20–B22, B24 | 6 |

*Algorithm for CCI implemented from the report: Lix L, Smith M, Pitz M, Ahmed R, Quon H, Griffith J, Turner D, Hong S, Prior H, Banerjee A, Koseva I, Kulbaba C. *Cancer Data Linkage in Manitoba: Expanding the Infrastructure for Research*. Winnipeg, MB: Manitoba Centre for Health Policy, 2016.

Figure S1: Predictors of in-hospital mortality in TTP hospitalizations


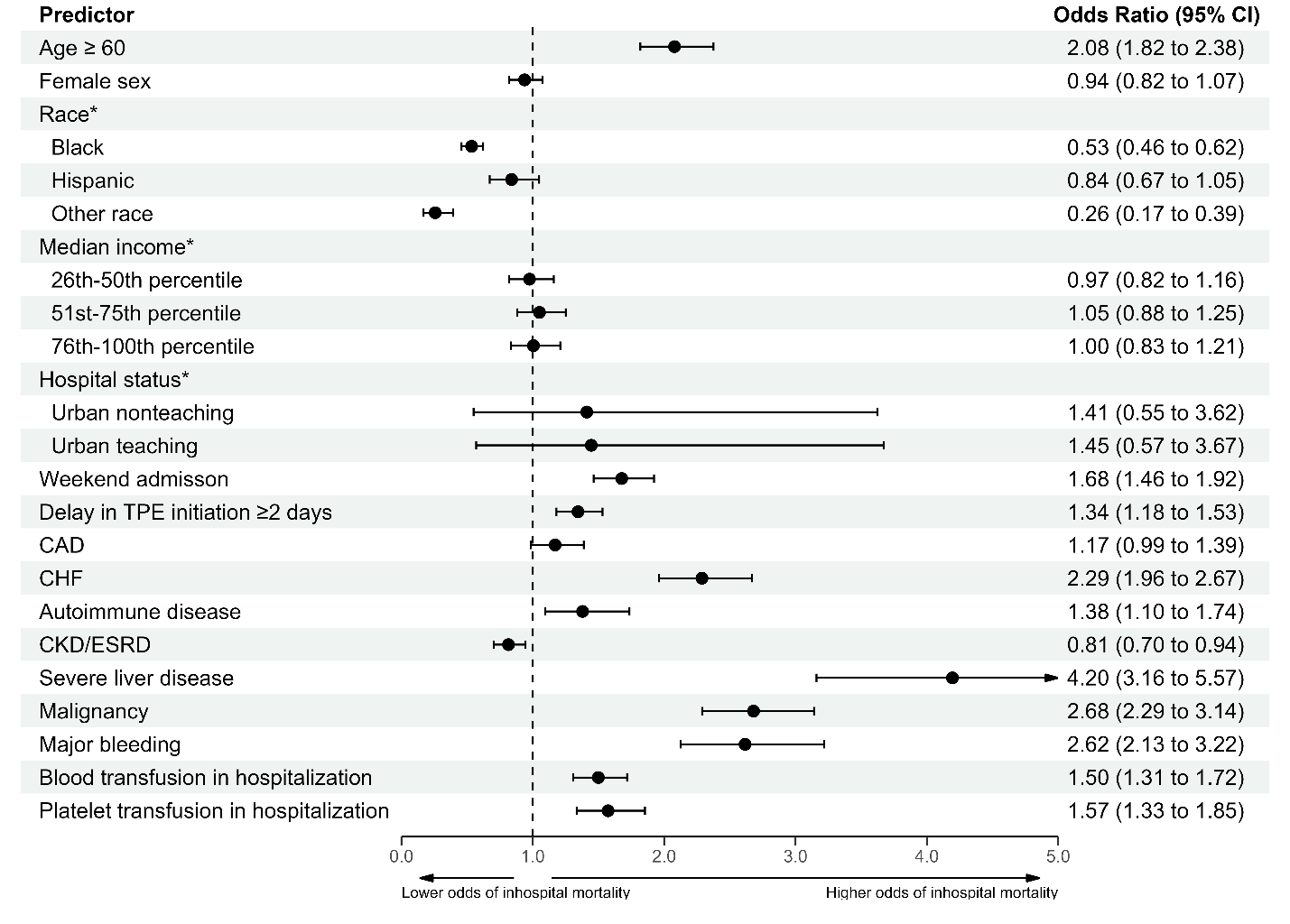

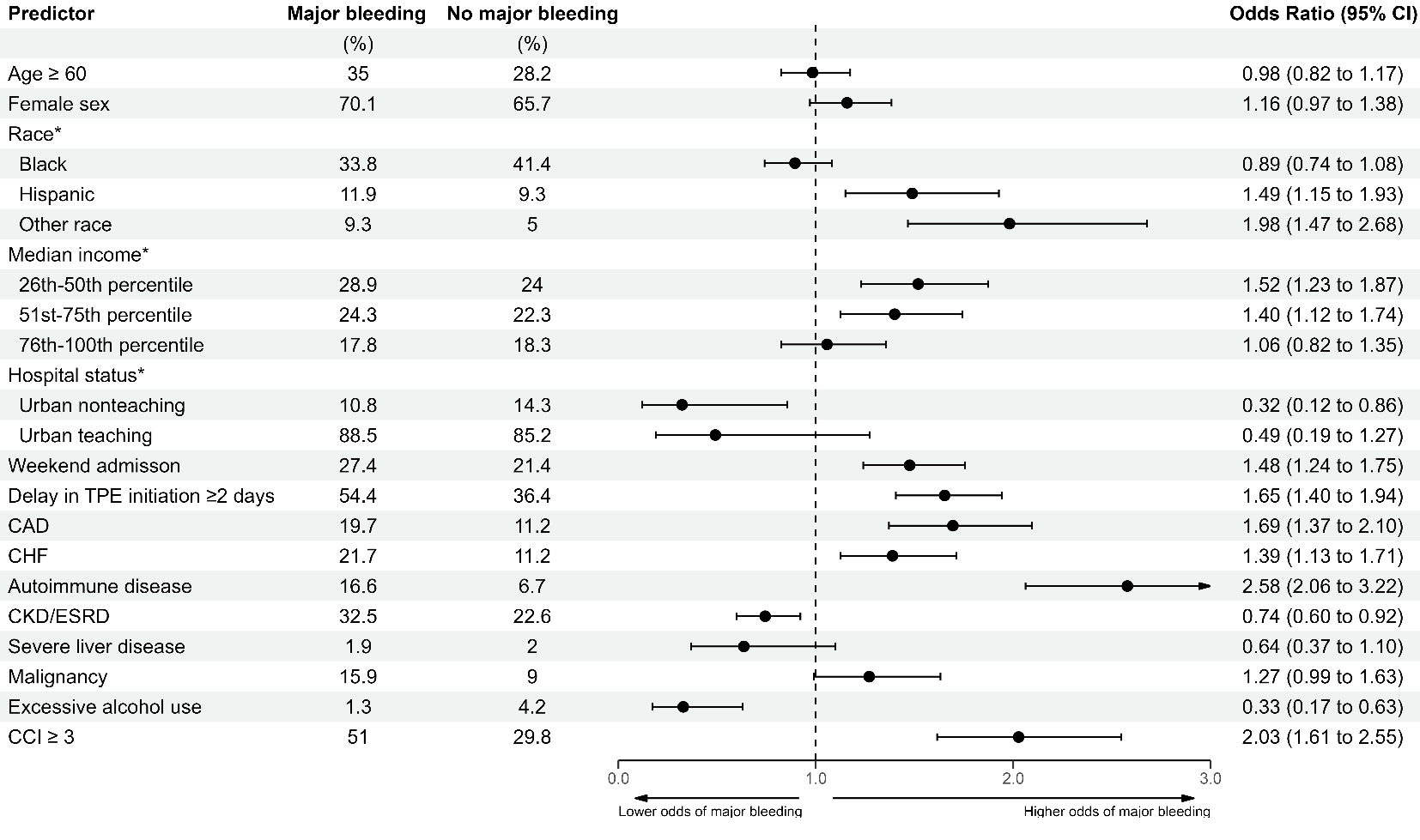


Figure S2: Predictors of major bleeding in TTP Hospitalization. Multivariable logistic regression was repeated without the long-term aspirin and anticoagulant variables due to concern that they might not capture medication use adequately.

Table S1: Bleeding types in hospitalizations with major bleeding

|  | | ***TTP hospitalizations with major bleeding***  ***(n = 157)*** |
| --- | --- | --- |
| ***Type of bleeding*** | | **N (%)** |
| ***GI Bleeding*** | | 13 (8.3) |
|  | Upper GI bleeding | 5 (3.2) |
|  | Lower GI bleeding | 7 (4.5) |
|  | Unspecified | 1 (0.6) |
| ***Hemoptysis*** | | 13 (8.3) |
| ***CNS bleeding*** | | 67 (42.7) |
|  | Subarachnoid hemorrhage | 19 (12.1) |
|  | Intracerebral hemorrhage | 39 (24.8) |
|  | Subdural hemorrhage | 14 (8.9) |
| ***Orbital or retinal hemorrhage*** | | 8 (5.1) |
| ***Hemoperitoneum*** | | 25 (15.9) |
| ***Hemothorax or Hemopericardium*** | | 55 (35.0) |
| ***Heavy menstrual bleeding/menorrhagia*** | | 4 (2.5) |
| ***Other mucosal bleeding*** | | 6 (3.8) |
| ***Skin wound or procedure-related bleeding*** | | 2 (1.3) |
| ***Hematuria*** | | 7 (4.5) |

Table S2: Characteristics of TTP hospitalizations with and without major bleeding

|  | | ***TTP hospitalizations***  ***(n=3,103)*** | | |
| --- | --- | --- | --- | --- |
|  | | ***With major bleeding***  ***(n=157)*** | ***Without major bleeding***  ***(n=2,946)*** | ***p-value*** |
| ***Age (Mean in years)*** | | 51.0 | 48.5 | 0.054 |
| ***Female — n (%)*** | | 110 (70.1) | 1935 (65.7) | 0.259 |
| ***Race*** ***— n (%)*** | |  |  | **0.04** |
|  | Caucasian | 68 (45.0) | 1245 (44.4) |  |
|  | African American | 51 (33.8) | 1162 (41.4) |  |
|  | Hispanic | 18 (11.9) | 260 (9.3) |  |
|  | Others | 14 (9.3) | 139 (5.0) |  |
| ***Income quartile — n (%)*** | |  |  | 0.330 |
|  | 1 | 44 (28.9) | 1020 (35.4) |  |
|  | 2 | 44 (28.9) | 693 (24.0) |  |
|  | 3 | 37 (24.3) | 643 (22.3) |  |
|  | 4 | 27 (17.8) | 528 (18.3) |  |
| ***Teaching status of the hospital — n (%)*** | |  |  | 0.481 |
|  | Rural hospital | 1 (0.6) | 16 (0.5) |  |
|  | Urban non-teaching | 17 (10.8) | 420 (14.3) |  |
|  | Urban teaching | 139 (88.5) | 2510 (85.2) |  |
| ***Weekend admission — n (%)*** | | 43 (27.4) | 630 (21.4) | 0.075 |
| ***Pregnancy/delivery — n (%)*** | | 4 (2.5) | 59 (2.0) | 0.559 |
| ***Comorbidities — n (%)*** | |  |  |  |
|  | Coronary artery disease | 31 (19.7) | 331 (11.2) | **0.001** |
|  | Congestive heart failure | 34 (21.7) | 331 (11.2) | **<0.001** |
|  | Autoimmune disease | 26 (16.6) | 196 (6.7) | **<0.001** |
|  | Chronic/End-stage renal disease | 51 (32.5) | 666 (22.6) | **0.004** |
|  | Mild to moderate liver disease | 7 (4.5) | 201 (6.8) | 0.248 |
|  | Severe liver disease | 3 (1.9) | 60 (2.0) | 1 |
|  | Cancer (localized or metastatic) | 25 (15.9) | 264 (9.0) | **0.003** |
|  | Excessive alcohol use | 2 (1.3) | 123 (4.2) | 0.091 |
|  | Obesity | 20 (12.7) | 527 (17.9) | 0.099 |
| ***Charlson comorbidity index — n (%)*** | |  |  | **<0.001** |
|  | 0 | 12 (7.6) | 1047 (35.5) |  |
|  | 1 | 38 (24.2) | 586 (19.9) |  |
|  | 2 | 27 (17.2) | 434 (14.7) |  |
|  | >=3 | 80 (51.0) | 879 (29.8) |  |
| ***Long term aspirin use — n (%)*** | | 7 (4.5) | 183 (6.2) | 0.372 |
| ***Long term anticoagulant use — n (%)*** | | 3 (1.9) | 110 (3.7) | 0.376 |
| ***Time to initiation of plasma exchange (days) — mean (95% CI)*** | | 5.2 (3.9-6.4) | 2.5 (2.3-2.6) | **<0.001** |
| ***Outcomes*** | |  |  |  |
|  | In-hospital mortality — n (%) | 38 (24.2) | 233 (7.9) | **<0.001** |
|  | Length of stay (days) — mean (95% CI) | 27.6 (23.2-32.0) | 14.4 (13.9-14.9) | **<0.001** |
|  | Received blood transfusion — n (%) | 91 (58.0) | 1041 (35.3) | **<0.001** |
|  | Received platelet transfusion — n (%) | 37 (23.6) | 396 (13.4) | **<0.001** |

Table S3: Comparison of baseline characteristics of unweighted vs weighted iTTP hospitalizations with bleeding

|  | | ***Unweighted iTTP hospitalizations***  ***(n=3,103)*** | ***Weighted iTTP hospitalizations***  ***(n=15,515)*** |
| --- | --- | --- | --- |
|  | | ***With bleeding***  ***(n=594) (19.1%)*** | ***With bleeding***  ***(n=2,970) (19.1%)*** |
| ***Age (Mean in years)*** | | 48.8 | 48.8 |
| ***Female — n (%)*** | | 400 (67.3) | 2000 (67.3) |
| ***Race*** ***— n (%)*** | |  |  |
|  | Caucasian | 259 (45.0) | 1295 (45.0) |
|  | African American | 213 (37.0) | 1065 (37.0) |
|  | Hispanic | 68 (11.8) | 340 (11.8) |
|  | Others | 35 (6.1) | 175 (6.1) |
| ***Income quartile — n (%)*** | |  |  |
|  | 1 | 201 (34.8) | 1005 (34.8) |
|  | 2 | 140 (24.3) | 700 (24.3) |
|  | 3 | 139 (24.1) | 695 (24.1) |
|  | 4 | 97 (16.8) | 485 (16.8) |
| ***Teaching status of the hospital — n (%)*** | |  |  |
|  | Rural hospital | 4 (0.7) | 20 (0.7) |
|  | Urban non-teaching | 78 (13.1) | 390 (13.1) |
|  | Urban teaching | 512 (86.2) | 2560 (86.2) |
| ***Weekend admission — n (%)*** | | 148 (24.9) | 740 (24.9) |
| ***Pregnancy/delivery — n (%)*** | | 8 (1.3) | 40 (1.3) |
| ***Comorbidities — n (%)*** | |  |  |
|  | Coronary artery disease | 84 (14.1) | 420 (14.1) |
|  | Congestive heart failure | 98 (16.5) | 490 (16.5) |
|  | Autoimmune disease | 60 (10.1) | 300 (10.1) |
|  | Chronic/End-stage renal disease | 160 (26.9) | 800 (26.9) |
|  | Mild to moderate liver disease | 24 (5.7) | 120 (5.7) |
|  | Severe liver disease | 23 (3.9) | 115 (3.9) |
|  | Cancer (localized or metastatic) | 74 (12.5) | 370 (12.5) |
|  | Excessive alcohol use | 33 (5.6) | 165 (5.6) |
|  | Obesity | 102 (17.2) | 510 (17.2) |
| ***Charlson comorbidity index — n (%)*** | |  |  |
|  | 0 | 148 (24.9) | 740 (24.9) |
|  | 1 | 119 (20.0) | 595 (20.0) |
|  | 2 | 96 (16.2) | 480 (16.2) |
|  | >=3 | 231 (38.9) | 1155 (38.9) |
| ***Long term aspirin use — n (%)*** | | 35 (5.9) | 175 (5.9) |
| ***Long term anticoagulant use — n (%)*** | | 25 (4.2) | 125 (4.2) |
| ***Time to initiation of plasma exchange (days) — mean (95% CI)*** | | 3.8 (3.3-4.4) | 3.8 (3.6-4.1) |
| ***Outcomes*** | |  |  |
|  | In-hospital mortality — n (%) | 100 (16.8) | 500 (16.8) |
|  | Length of stay (days) — mean (95% CI) | 19.9 (18.3-21.5) | 19.9 (19.2-20.6) |
|  | Received blood transfusion — n (%) | 290 (48.8) | 1450 (48.8) |
|  | Received platelet transfusion — n (%) | 126 (21.2) | 630 (21.2) |
